# Parameters of Iliopsoas Plane Immediately Caudal to the Indirect Tendon of Rectus Femoris in Axial Plane Measured on Magnetic Resonance Images in an Adult Eastern Asian Population

**DOI:** 10.3390/healthcare11010069

**Published:** 2022-12-26

**Authors:** Shang-Ru Yeoh, Yen Chou, Jin-Han Yang, Ching-Wei Chuang, Shun-Ming Chan, Se-Yi Chen, Jin-De Hou, Jui-An Lin

**Affiliations:** 1 Department of Anesthesiology, Wan Fang Hospital, Taipei Medical University, Taipei 116, Taiwan; 2 Center for Regional Anesthesia and Pain Medicine, Wan Fang Hospital, Taipei Medical University, Taipei 116, Taiwan; 3 Department of Medical Imaging, Fu Jen Catholic University Hospital, New Taipei City 243, Taiwan; 4 Medical Imaging and Artificial Intelligence Laboratory, Far Eastern Memorial Hospital, New Taipei City 220, Taiwan; 5 Department of Internal Medicine, Taipei Veterans General Hospital, Taipei 112, Taiwan; 6 Graduate Institute of Clinical Medicine, College of Medicine, Taipei Medical University, Taipei 110, Taiwan; 7 Department of Anesthesiology, Tri-Service General Hospital and National Defense Medical Center, Taipei 11490, Taiwan; 8 Department of Neurosurgery, Chung Shan Medical University Hospital, Taichung 40201, Taiwan; 9 School of Medicine, Chung Shan Medical University, Taichung 40201, Taiwan; 10 Division of Anesthesiology, Hualien Armed Forces General Hospital, Hualien 97144, Taiwan; 11 Department of Anesthesiology, School of Medicine, National Defense Medical Center, Taipei 11490, Taiwan; 12 Center for Regional Anesthesia and Pain Medicine, Chung Shan Medical University Hospital, Taichung 40201, Taiwan; 13 Department of Anesthesiology, School of Medicine, Chung Shan Medical University, Taichung 40201, Taiwan; 14 Department of Anesthesiology, Chung Shan Medical University Hospital, Taichung 40201, Taiwan; 15 Department of Anesthesiology, School of Medicine, College of Medicine, Taipei Medical University, Taipei 110, Taiwan

**Keywords:** hip, iliopsoas plane, pain, parameters, quadriceps muscle: rectus femoris, tendons: indirect, radiofrequency ablation

## Abstract

The iliopsoas plane (IP) is a fascial plane deep to the iliopsoas complex and is the target of several novel ultrasound-guided analgesic interventions for hip. Currently, limited information is known about its parameters. From the pelvic magnetic resonance (MR) images of an adult Eastern Asian population (n = 49), the IP width, depth, and needle-beam angle in the axial plane immediately caudal to the level of indirect tendon of rectus femoris (RF) were found to be 10.7 ± 1.6 mm, 48.5 ± 15.5 mm, and 84.2 ± 8.2 degrees, respectively. There was a statistically significant difference in the age categories for IP width, and older patients seemed to have wider IP. Our data may provide applications for the technical modification of ultrasound-guided iliopsoas plane block (IPB) in acute hip pain management and the future development of ultrasound-guided single-needle-entry radiofrequency neuroablation in chronic hip pain management.

## 1. Introduction

With advances in knowledge on the iliopsoas plane (IP) [1] and its neural contents [2], ultrasound-guided analgesic interventions for hip performed at the level of capsular ligaments of hip, such as iliopsoas plane block (IPB) [3] and the anterior approach to hip pericapsular neurolysis [4,5], have recently been developed. However, little is known about the IP parameters and their interindividual variations. Therefore, the objective of this study is to provide a preliminary IP quantification for the subsequent technical improvement of these novel ultrasound-guided techniques. For example, when performing IPB or anterior hip pericapsular injection, the optimal final needle tip position relative to the iliopsoas tendon (IT) in an ultrasound transverse scan can be more objectively determined based on IP width to avoid either inadvertent injection of the iliopectineal bursa that is deep to the IT or intramuscularly into the iliacus minor muscle (IM) [6]. In this study, we measured IP width, depth, and needle-beam angle at the level immediately caudal to the appearance of the indirect (reflected) tendon of rectus femoris (RF) in the axial plane of the pelvic magnetic resonance (MR) images acquired from an adult Eastern Asian population.

## 2. Methods

**Figure 1:**
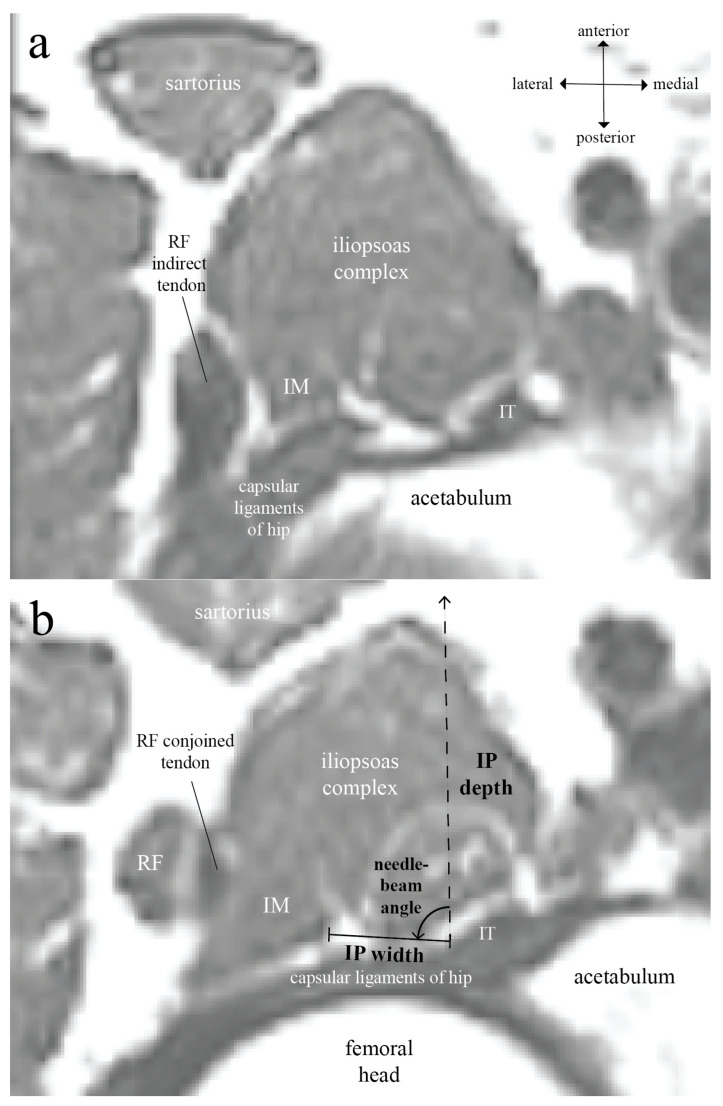
Axial plane of T2-weighted magnetic resonance (MR) images of the human right hip: (**a**) At the level where rectus femoris (RF) indirect tendon appears and extends anteroposteriorly towards acetabular rim; (**b**) At the level 10–15 mm caudal to Figure 1a, where the RF indirect and direct tendon merge into its conjoined tendon, the iliopsoas plane (IP) width, depth, and needle-beam angle were measured. The anterior skin surface used for IP depth measurement is not shown.

MR images from patients 18 years or older were retrieved retrospectively from the image database of a single institute (Far Eastern Memorial Hospital, New Taipei City, Taiwan). Only the MR studies of pelvis/bilateral hips performed for indications other than hip pathologies or related complaints were included. All MR studies were de-identified and anonymized in accordance with the standard practices of the HIPAA Privacy Rule before analysis. For each enrolled subject, we also acquired demographic information including age, gender, body height, and body weight for subgroup analysis. For IP width measurement, we uniformly used the default musculoskeletal windowing provided by the open-sourced MicroDicom DICOM viewer (software version 2022.2, MicroDicom, Sofia, Bulgaria) to best distinguish different components of the iliopsoas complex and the capsular ligaments of hip. This allows us to measure the IP widths more accurately and reproducibly (Figure 1). Patients with MR images in which RF and its tendons were not fully acquired were excluded. Patients with MR images in which IM borders were not radiographically identifiable were also excluded.

**Figure 2:**
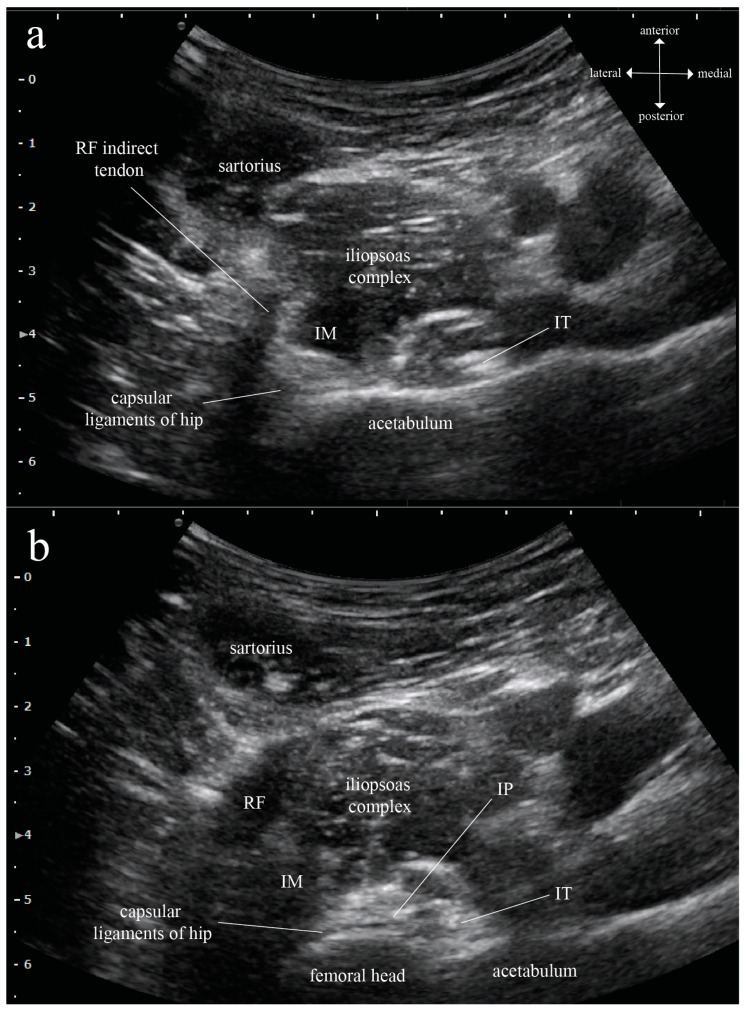
Ultrasound transverse scan of the human right hip with a curvilinear probe: (**a**) At the level corresponding to Figure 1a, the rectus femoris (RF) indirect tendon is an easily identifiable, elongated hypoechoic shadow lateral to the iliopsoas complex that can be used as a startup sonographic landmark to identify the iliopsoas plane (IP) at the level of iliopsoas plane block (IPB) and anterior hip pericapsular neurolysis. Under ultrasound, the iliopsoas complex and the capsular ligaments of hip are both hypoechoic, while iliopsoas tendon (IT) is a hyperechoic ovoid structure situated at the medial corner of iliopsoas complex. (**b**) At the level corresponding to Figure 1b, in between the iliopsoas complex and capsular ligaments of hip, the IP appears as a fuzzy, hyperechoic layer. The IM medial border is, in general, very difficult to identify sonographically, making the lateral margin of IP obscure and hard to define on ultrasound.

Since the MR images give far better structural contrast than ultrasound images and are generally not subject to operator-related variations, anatomic landmarks that can be reliably identified on both MR and ultrasound images are needed to translate the findings on MR images into ultrasound-guided techniques. To locate the level where IPB [1,3] and anterior hip pericapsular neurolysis [4,5] are performed in the transverse section on the ultrasound, we found the indirect (reflected) tendon of RF to be a very useful anatomic landmark. From the level where the RF indirect tendon is identifiable in an MR axial plane (Figure 1a), when going caudally, the RF indirect tendon can be seen joining its direct (straight) tendon to merge into the RF conjoined tendon (Figure 1b). The level where the RF indirect tendon is identifiable in the axial plane corresponds to the level immediately cephalad to where IPB and anterior hip pericapsular neurolysis are performed. Because the RF indirect tendon extends anteroposteriorly to attach along the acetabular rim [7], it goes almost perpendicular to the ultrasound probe in the transverse scan, creating a prominent elongated hypoechoic shadow just lateral to the iliopsoas complex (Figure 2). Therefore, we adopted the RF indirect tendon as a startup landmark to approximate the level where IP parameters were measured on the MR images. The distance of the caudal shift, with which the RF indirect and direct tendon merge into the RF conjoined tendon, was measured on the MR images to be 10–15 mm. At the level after the caudal shift, IP width, depth, and needle-beam angle were measured on the right side of hip. The IP width is defined as the distance of a line drawn between the medial border of IM and lateral border of IT, the IP depth as the distance of a vertical line drawn between the anterior skin surface and the IT lateral border, and the needle-beam angle as the angle between these two lines (Figure 1b). All parameters were measured manually using MicroDicom’s built-in measurement functions.

The difference between groups was analyzed according to the following methods: Student’s *t*-test was used to analyze the mean difference between two groups, and one-way analysis of variance (ANOVA) was used when there was equal or more than three groups. A Tukey post hoc test was used for multiple comparison analysis. Continuous variables are presented as mean and standard deviation. Factors with *p* < 0.05 was considered as statistically significant. Statistical analysis was performed with SPSS version 25 (SPSS Inc., Chicago, IL, USA).

## 3. Results

T2-weighted axial plane pelvic MR imaging studies of 30 male and 30 female patients were retrospectively retrieved. Eleven patients were excluded due to incomplete imaging of RF and its tendons (n = 8), poor image quality of the iliopsoas complex and IM (n = 1), and duplication (n = 2). For the enrolled patients (n = 49), mean age was 61.8 years old, and mean BMI was 26.2 kg/m^2^. IP width, depth, and needle-beam angle were found to be 10.7 ± 1.6 mm, 48.5 ± 15.5 mm, and 84.2 ± 8.2 degrees, respectively (mean ± Std). We were unable to measure the IP depth of one enrolled patient because the skin surface exceeded the field of view on the MR study due to thick subcutaneous fat.

For IP width, there was a statistically significant difference only between the age (*p*= 0.023) but not the gender nor BMI categories, and a Tukey post hoc test revealed that individuals aged 70 years and older had a significantly wider IP than individuals aged below 50 years old (*p* = 0.017). For IP depth, a statistically significant difference was found between all group pairs in the BMI (*p* < 0.001) but not the age or gender categories. For needle-beam angle, there was no statistically significant difference between the age, gender, or BMI categories (Table 1). IP width seemed to increase with age, but the correlation model between these two parameters by our linear regression analysis could not fully establish a trend (R square = 0.132). On the other hand, stronger correlation was found between IP depth and the BMI categories (R square = 0.563), with which IP depth increases with BMI (Table 2).

## 4. Discussion

IP is a fascial plane deep to the iliopsoas complex that is bordered laterally by IM and medially by IT [1,3,6]. In the axial plane on the MR images, at the level that is 10–15 mm caudal to where the RF indirect and direct tendon merge into its conjoined tendon, the IP width was about 10 mm. IP width seemed to increase with age, and the IP was 2.5 mm wider in patients aged over 70 years old than those below 50 years old. However, a clear statistical correlation between IP width and age could not be found, possibly due to our relatively small sample size. Age-related property changes in joint and muscle anatomy may be at play but require further exploration. The needle trajectory parallel to the IP almost forms a right angle with the direction of ultrasound beam.

There are at least two practical applications of these data to the ultrasound-guided analgesic interventions. Firstly, the original IPB described by Nielsen et al. [1,3] is an inferolateral-to-superomedial oblique in-plane injection into the IP with no objective information on how far away the needle tip should be relative to IT. Anatomically, IP is a narrow space bordered laterally by IM and medially by IT, and the more caudal it goes, the narrower the space is [6]. Now, since we know that the IP width is only about 10 mm at the level where IPB is performed, the technique therefore warrants some modification to ensure a consistent and precise IP injection. The lateral-to-medial in-plane approach of the anterior hip pericapsular infiltration firstly described by Sasaki et al. [4] allows visualization of all the relevant structures during IP injection. However, the needle tip was advanced all the way through until it was buried deep to the IT [4,5] as in the pericapsular nerve group block [8], which may cause accidental iliopectineal bursal injection [6]. For a “true” ultrasound-guided IP injection in the transverse scan, we propose that the needle tip be left inside the hyperechoic layer between the iliopsoas complex and capsular ligaments of hip (i.e., IP, see Figure 2) within 10 mm lateral to IT. Moreover, IT should be used as a sonographic proxy to approximate the lateral border of IM, which is generally not identifiable on an ultrasound. Because 10 mm is quite a narrow margin for an ultrasound-guided procedure, a test spread with normal saline before the procedural injectate is also recommended. In our institution, we have so far utilized this modified technique successfully in two outpatient cases with chronic hip pain. Immediate pain relief with improved range of motion and no decreased quadriceps muscle strength were achieved with 3 ml of 0.3% ropivacaine in both patients, and the analgesic effects lasted for about 2 weeks.

Secondly, our result may also be applied to developing a new way of ultrasound-guided neuroablation for chronic hip pain. Nociceptors of the hip joint capsule are primarily distributed in the anterior hip capsule, with their parent nerves coming from femoral, obturator, and the accessory obturator nerve [9]. Because both the high and low femoral articular branches (and perhaps also the accessory obturator nerve) traverse the IP before innervating the anterior hip capsule [2,10], interception of the nociceptive signals transmitted via these hip articular branches within IP forms the basis of IPB as a motor-sparing hip block. Since the IP immediately distal to the RF indirect tendon is just about 10 mm wide, single-needle-entry radiofrequency neuroablation of IP with ultrasound guidance alone may become amenable. However, the analgesic effects of ablating this narrow IP may be dependent on the proportion of the femoral articular branches to hip (and perhaps also the accessory obturator nerve) that passes through the plane after leaving the psoas valley [2,6]. In addition, because the needle-beam angle is close to 90 degree, in-plane insertion of the ablation needle parallel to the IP gives a near-optimal trajectory for needle visualization on ultrasound.

The major limitation of this study is the observer bias in determining the anatomical landmarks on MR images, for example, the IM medial border and IT lateral border during IP width measurements. Nonetheless, the bias may become clinically insignificant when put into the context of ultrasound-guided procedures, of which the procedural success is widely known to be dependent on the operator’s ability to recognize anatomical structures and finding/controlling the needle tip under ultrasound.

## 5. Conclusions

IP width is approximately 10 mm and the needle-beam angle is close to 90 degrees in the axial plane at the level 10–15 mm caudal to the RF indirect tendon on the MR images in an adult Eastern Asian population. The data can provide applications to the technical modification of IPB for more accurate needle tip placement and perhaps the future development of single-needle-entry neuroablation with ultrasound guidance only.

## Figures and Tables

**Table 1:** Iliopsoas plane (IP) width, depth, and needle-beam angle (mean ± Std) shown by subgroups in the age, gender, and body mass index (BMI) categories.

	N (%)	IP Width (mm)	IP Depth (mm)	Needle-Beam Angle (Degrees)
Age, years/old				
<50	6 (12)	9.5 ± 1.2	42.1 ± 9.9	86.6 ± 1.0
50–59	11 (23)	10.5 ± 1.2	54.9 ± 24.3	84.2 ± 8.5
60–69	21 (44)	10.7 ± 1.6	48.3 ± 12.5	84.5 ± 9.2
>70	10 (21)	12.0 ± 1.9	45.5 ± 11.0 ^c^	81.9 ± 5.4
*p* value ^a^		0.023	0.372	0.754
Gender, n				
Female	21 (44)	10.3 ± 1.4	53.5 ± 21.1	83.6 ± 8.2
Male	27 (56)	11.1 ± 1.8	44.5 ± 7.6 ^c^	84.6 ± 8.6
*p* value ^b^		0.076	0.051	0.677
BMI, kg/m^2^				
18.5–24.9	19 (40)	11.2 ± 1.6	38.9 ± 5.8	85.4 ± 9.5
25–29.9	22 (46)	10.5 ± 1.7	50.1 ± 8.0	83.4 ± 8.1
30–39	7 (14)	10.5 ± 1.3	73.2 ± 27.8 ^c^	83.3 ± 6.0
*p* value ^a^		0.323	<0.001 ^d^	0.736

BMI: body mass index. a. Calculated by one-way ANOVA. b. Calculated by Student’s t test. c. Depth was not measurable in one patient due to insufficient field of view. d. *p* < 0.001 was found between all group pairs.

**Table 2:** Linear regression analyses of IP width and IP depth with age and BMI, respectively.

R Square (Adjusted R)		Sig
0.150 (0.132)	IP width = 6.977 + 0.061 ∗ age	0.007
0.573 (0.563)	IP depth = −31.24 + 3.064 ∗ BMI	<0.001

For more details can be found at Appendix A.

**Figure A1:**
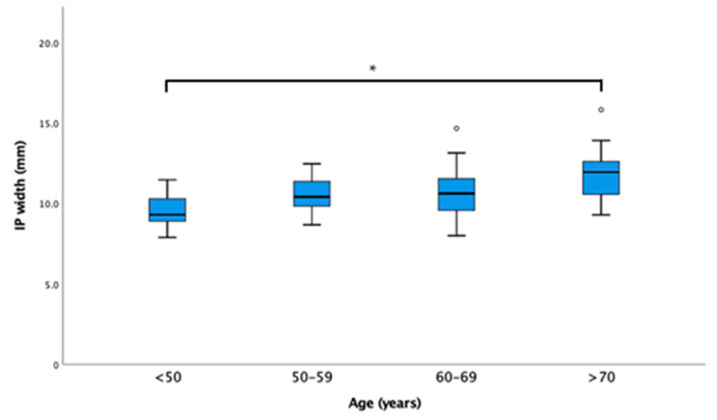
IP width with the age subgroups as box-plots. *: statistically significant be-tween groups; °: outliers.

**Figure A2:**
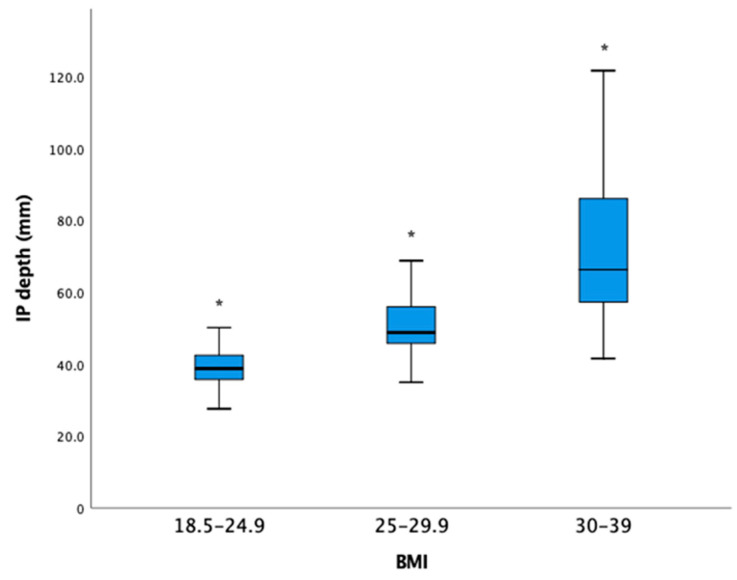
IP depth with the BMI subgroups as box-plots. BMI: body mass index; IP: iliopsoas plane. *: statistically significant between groups.

## Data Availability

Not applicable.

## Appendix B

To facilitate reading, abbreviations used in this article are listed here in alphabetical order. BMI: body mass index; IM: iliacus minor; IP: iliopsoas plane; IPB: iliopsoas plane block; IT: iliopsoas tendon; MR: magnetic resonance; RF: rectus femoris.
